# Co-Influence of Nanofiller Content and 3D Printing Parameters on Mechanical Properties of Thermoplastic Polyurethane (TPU)/Halloysite Nanotube (HNT) Nanocomposites

**DOI:** 10.3390/nano13131975

**Published:** 2023-06-29

**Authors:** Wendy Triadji Nugroho, Yu Dong, Alokesh Pramanik, Zhixiao Zhang, Seeram Ramakrishna

**Affiliations:** 1 School of Civil and Mechanical Engineering, Curtin University, P.O. Box U1987, Perth, WA 6845, Australia; w.nugroho1@postgrad.curtin.edu.au (W.T.N.); alokesh.pramanik@curtin.edu.au (A.P.); 2 School of Materials Science and Engineering, Hebei University of Engineering, Handan 056038, China; zhixiao351@hebeu.edu.cn; 3 Department of Mechanical Engineering, National University of Singapore, Singapore 117575, Singapore; seeram@nus.edu.sg

**Keywords:** thermoplastic polyurethane (TPU), halloysite nanotubes (HNTs), Taguchi design of experiments (DoEs), fused deposition modelling (FDM), mechanical properties, material characterisation

## Abstract

Thermoplastic polyurethane (TPU) belongs to a polyurethane family that possesses an elongation much higher than 300%, despite having low mechanical strength, which can be overcome by incorporating clay-based halloysite nanotubes (HNTs) as additives to manufacture TPU/HNT nanocomposites. This paper focuses on the co-influence of HNT content and 3D printing parameters on the mechanical properties of 3D printed TPU/HNT nanocomposites in terms of tensile properties, hardness, and abrasion resistance via fused deposition modelling (FDM). The optimum factor-level combination for different responses was determined with the aid of robust statistical Taguchi design of experiments (DoEs). Material characterisation was also carried out to evaluate the surface morphology, nanofiller dispersion, chemical structure, thermal stability, and phase behaviour corresponding to the DoE results obtained. It is evidently shown that HNT level and infill density play a significant role in impacting mechanical properties of 3D-printed TPU/HNT nanocomposites.

## 1. Introduction

TPU is one of most popular polyurethanes (PUs), and is synthesised using soft and hard polymeric segments in an alternating manner. Soft segments with a low glass transition temperature (*T_g_*) are essential for yielding continuous matrices in polymer nanocomposites with great flexibility at low temperatures, as opposed to the hard segments, which are inclined to undergoing self-assembly into domains via a crosslinking effect [1]. Generally speaking, physical crosslinks appear to be reversible, which implies that soft and hard segments possess the characteristics of soft segments, in order to create a homogenous mixture beyond *T_g_* [1]. This unique characteristic depends on morphological structures associated with specific chemical structures and processing conditions. Accordingly, TPU has been widely used in many applications such as coatings [2], biomaterials [3], structural foams [4], scaffolds [5], finger orthosis [6], biomedical devices [7], biomimicked skeletal muscle actuators [8], strain sensors [9], wearable devices [10], as well as stretchable organic thermoelectric generators [11].

Neat TPU undergoes very large elongation up to approximately 400% despite its low mechanical strength. The addition of nanoparticles as reinforcing additives enables the mechanical strength to be improved instead. Accordingly, 3D structures such as scaffolds, flexible sensors and wearable devices are often generated using TPU composites [12]. Multi-walled carbon nanotubes (MWCNTs), as one of most popular nanoparticle types, can be employed as reinforcements to enhance the tensile strength and Young’s modulus of TPU. In particular, it has been reported that the tensile strength and Young’s modulus of corresponding composites can be increased by approximately 39 and 49% with the addition of 3 wt% MWCNTs in TPU matrices [13]. However, the tensile strength of these composites dropped by 72.24% with the incorporation of 5 wt% MWCNTs, although Young’s modulus was still improved by approximately 20.17% [11]. Another type of nanoparticles, montmorillonites (MMTs), can enhance the hydrophilicity, dye absorption capacity and antifouling property of fibrous polyurethane (PU) membranes. Such nanocomposite materials reinforced with 20 wt% MMTs yielded a smaller water contact angle of 57° when compared with neat fibrous PU with a contact angle of 117°, along with high water flux and oil rejection, and they are thus able to meet the requirement of antifouling membranes used for waste-water treatment [14]. Maamoun et al. [15] utilised HNTs to promote the sound absorption capability of PU. With the embedding of 1 wt% HNTs, the sound absorption coefficient of nanocomposite materials was improved, as evidenced by the typical shift from a high-frequency to mid-frequency range, as opposed to unfilled PU. However, there was no significant effect on sound absorption behaviour when increasing the HNT content from 2 to 5 wt%. This phenomenon may be induced by the adverse effect of high nanoparticle concentration within PU matrices, resulting in the deterioration of urethane stability.

Additive manufacturing (AM), one of most advanced manufacturing techniques, is capable of producing 3D structures in complex geometry based on the fundamental principle of material deposition in a layer-by-layer manner for the manufacture of final structures. More remarkably, AM technologies have been identified as significantly reducing the amount of material waste, as compared to conventional subtractive manufacturing such as computer numerical control (CNC) machining [16]. In particular, fused deposition modelling (FDM) is among most popular AM techniques that is specifically used for plastics and polymer composites, as well as more recently polymer nanocomposites in the context of low-cost manufacturing strategies [17]. FDM depends primarily on computer-aided design (CAD) to generate 3D models, which can be converted into Standard Tessellation Language (STL) format, and further read by the slicing software, which includes Apex 1.8.4 [18], Makerbot Print 4.10.1 [19] and Cura 4.3 [20]. Kokcu et al. [21] applied this advanced technology to manufacture polylactic acid (PLA)/HNT nanocomposite scaffolds at HNT contents of 1–5 wt%. Specifically, it was found that the incorporation of 3 wt% HNTs could significantly enhance the tensile strength, compressive strength and flexural strength of nanocomposite scaffolds by 124, 145 and 41% respectively. Lv et al. [22] implemented the FDM technique to successfully prepare flexible composites based on graphene-modified polyolefin elastomer (POE) in possession of well-tailored porous structures for the applications of electromagnetic interference shielding and thermal management. With the inclusion of 10.93 vol% graphene nanoplatelets (GNPs), the electromagnetic shielding efficiency of printed nanocomposite structures reached 35 dB and the maximum thermal conductivity became 4.3 W·m^−1^·K^−1^, which was about 1600% higher than that of neat POE. On the other hand, Ghaziof et al. [23] prepared poly *ε*-caprolactone (PCL)/gold nanoparticle nanocomposite scaffolds with the aid of the FDM technique. It was shown that the addition of 0.5 wt% gold nanoparticles increased the compressive strength and electrical conductivity of the scaffolds by 9.1 and 25% respectively, suggesting that such scaffolds might be a potential material candidate for cardiovascular applications.

Additively manufactured TPU/HNT nanocomposite materials using FDM have been widely investigated in several studies [24,25,26] evaluating the effect of HNTs on the mechanical properties and cytotoxicity of TPU/HNT nanocomposites. This led to 26 and 50% increases in tensile strength and elongation at break with the inclusion of 2 wt% HNTs relative to those of neat TPU. Furthermore, cytotoxicity tests, performed in association with 3-[4,5-dimethylthiazol-2-yl]-2.5-diphenyltetrazolium bromide in order to assess materials’ toxicity level as potential biomaterials, indicated a lack of cytotoxicity in the tested materials towards normal human body cells. Manuhaki et al. [25] applied a melt-compounding method using a laboratory-scale internal mixer to prepare TPU/HNT nanocomposites, resulting in the improvements of tensile strength, tensile modulus, and toughness of 29.82, 73.65 and 62.81% with the inclusion of 4.2 wt% HNTs. The incorporation of HNTs into TPU matrices was also observed to improve the thermal stability and sound absorption in this nanocomposite system. The addition of 1 wt% HNTs made it possible to increase the *T_g_* of corresponding nanocomposites, since rigid nanofillers generally induce restricted mobility of soft segments in molecular chains. Moreover, the inclusion of 1 wt% HNTs appeared to shift the mid-frequency range instead of its high-frequency counterpart, thus offering better sound absorption, as compared to neat TPU [15]. Prasanthi et al. [26] reported that the sorption capacity of PU/HNT/fluorinated graphene (FG) nanocomposite sponges was in the range of 38–62 g·g^−1^, with remarkable recyclability under static and turbulent conditions, as well as excellent corrosive and thermal stabilities.

Nonetheless, the determination of optimal factor-level combination of additively manufactured TPU/HNT nanocomposites via FDM and Taguchi design of experiments (DoEs) have rarely been investigated in a systematic manner, particularly with regard to mechanical properties, which is the major focus of this study. This paper investigates the combined effect of nanofillers and 3D printing parameters on key mechanical properties of TPU/HNT nanocomposites as a novel perspective on the systematic development of additively manufactured polymer nanocomposites to achieve the desired properties and specific applications of end users. It also paves the way to establishing a robust processing–structure–property relationship for 3D printed TPU/HNT nanocomposites for widespread applications.

## 2. Materials and Methods

### 2.1. Materials

**Figure 1:**
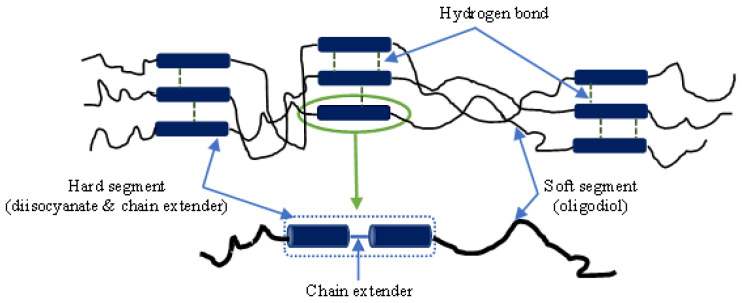
Chemical structure of TPU [30].

MM-4520 TPU (*T_g_* = 45 °C and melt viscosity of 3310 Pa·s at 215 °C) was supplied by SMP Technologies Inc. [27], Tokyo, Japan as the base polymer, while HNTs, as the additives, were donated by Imerys Ceramics [28], Matauri Bay, New Zealand. Dimethylformamide (DMF) was purchased from ChemSupply, Gillman, Australia as a chemical solvent to dissolve TPU. All materials were used without modification. The chemical structure of TPU used in this study is illustrated in Figure 1. Hard segments of TPU generally comprise diisocyanate and chain extender while soft components contain oligodiol. Meanwhile, HNTs are classified as clay-based nanomaterials, which belong to Kaolin family in possession of typical hollow and tubular structures and high aspect ratios with a chemical formula of Al_2_Si_2_O_5_(OH)_4_·nH_2_O. Generally speaking, the inner and outer diameter of well-dispersed HNTs can reach 1–30 nm and 30–50 nm, along with a length between 100 and 2000 nm [29]. The physical and mechanical properties of MM-4520 and HNTs are listed in Tables S1 and S2 (Supporting Information).

### 2.2. Preparation of TPU/HNT Nanocomposites

**Figure 2:**
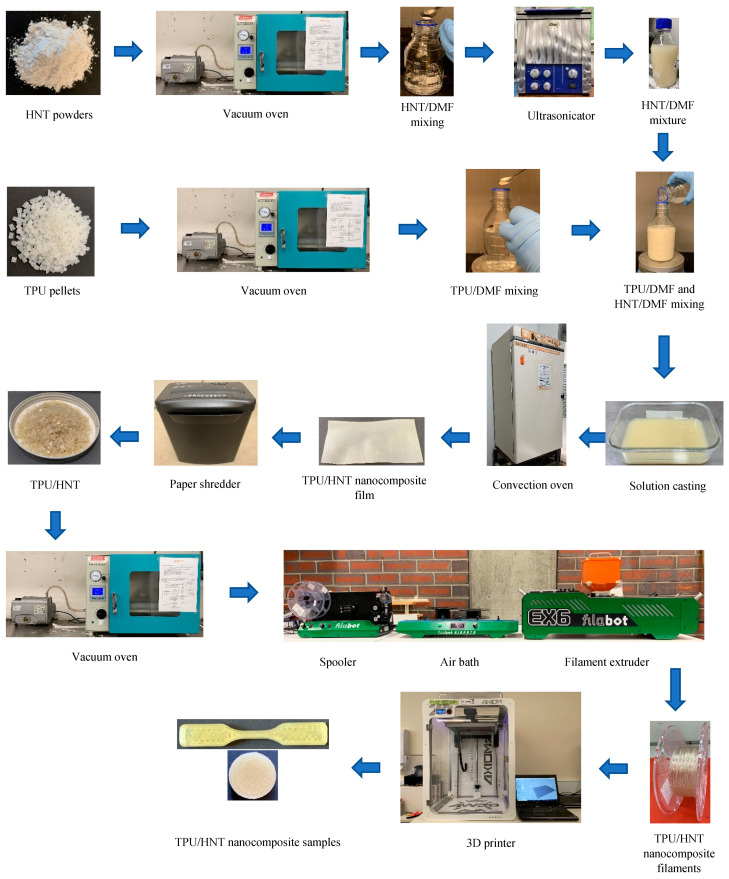
Flow chart of the manufacturing process of TPU/HNT nanocomposite films, extruded filaments and 3D-printed samples.

HNT dispersion in TPU matrices is key to obtaining the desired mechanical properties. The driving force for enhancing the dispersibility of HNTs in this study comprises material processing steps involving the drying process, the solvent and mixing processes. The drying process is required to reduce the moisture content of raw materials so that they can be easily separated when undergoing the mixing process, which is particularly the case for HNT powders. It is recommended that a solvent be appropriately selected in order to facilitate dissolving TPU pellets. Meanwhile, the mixing method should be carefully chosen to ensure the effective dispersion of HNTs within polymer matrices. As such, this study utilised DMF as a chemical solvent, and heating, stirring and ultrasonication were carried out as major mixing techniques. TPU/HNT nanocomposites were prepared using multiple stages of material manufacturing, including solution casting, extrusion and FDM, as shown in Figure 2. In the first stage of solution casting to produce TPU/HNT nanocomposite films, both TPU pellets and HNT powders were dried using a vacuum oven at 80 °C for 4 and 8 h respectively according to previous studies [27,31] in order to minimise the moisture effect. The required amount of HNTs was dispersed in DMF solvent at a weight ratio of 1:30 [32] using an ultrasonicating bath at the power intensity of 90% and a frequency of 25 kHz for a sonication time of 1 h [33]. Conversely, TPU/DMF mixture was blended at a weight ratio of 1:8, as suggested by Rosales et al. [32]. DMF was initially heated and processed using a magnetic stirrer at an elevated temperature of 300 °C with a rotor speed of 700 rpm prior to mixing TPU pellets and DMF. TPU pellets were steadily added in small amounts to the beaker to prevent the typical issue of material sedimentation occurring before all TPU pellets had been fully dissolved. Furthermore, HNT/DMF mixture was poured into its TPU/DMF counterpart in solution form while being subjected to continuous stirring and heating. After 30 min, final TPU/HNT/DMF mixture was cast onto a glass petri dish, and further heated in an oven for solvent removal at 100 °C for 24 h. TPU/HNT nanocomposite films were manufactured according to previous studies [34,35], which were then stored in a silicon gel-containing desiccator prior to material characterisation and testing.

During the second stage of the extrusion process, a single-screw filament extruder Filabot EX6 (screw diameter: 16 mm; L/D ratio: 24), purchased from the Filabot company, Montpelier, VT, USA, was employed to fabricate TPU/HNT nanocomposite filaments at a screw speed of 50 rpm. Four temperature zones in the extrusion process consist of individual temperature settings of 165, 165, 165 and 45 °C for the front, middle, back and feed zones respectively. TPU/HNT nanocomposite films were initially shredded and chopped into small flakes with a length, width and thickness of 4.30 ± 0.5, 3.00 ± 0.1 and 0.45 ± 0.1 mm accordingly. They underwent a drying process in a vacuum oven at 80 °C for 4 h to remove the moisture. Fabricated filaments were spooled, dried and stored in airtight containers with silica gels. The same procedure was followed with respect to TPU filaments without shredding and chopping. The extrusion parameters of the Filabot extruder were set according to those presented in Table S3 (Supporting Information).

During the final stage, type V dog-bone and cylindrical samples of TPU and TPU/HNT nanocomposites were prepared using FDM technique according to ASTM D638 [36] and ASTM D5963 [37] standards. An Axiom 20 3D printer and APEX 1.7.4 slicer software, supplied by Airwolf 3D company, Costa Mesa, CA, USA, were employed to manufacture final printed samples. The impacts of material formulation and 3D printing parameters such as HNT level, nozzle temperature, print speed, infill density and layer height on tensile properties, hardness and abrasion resistance of TPU/HNT nanocomposites were holistically investigated. Table 1 lists material formulation and 3D printing parameters selected based on our previous work [38] via Taguchi design of experiments (DoEs). The other 3D printing parameters were fixed in this study, as shown in Table 2.

### 2.3. Mechanical Testing

**Figure 3:**
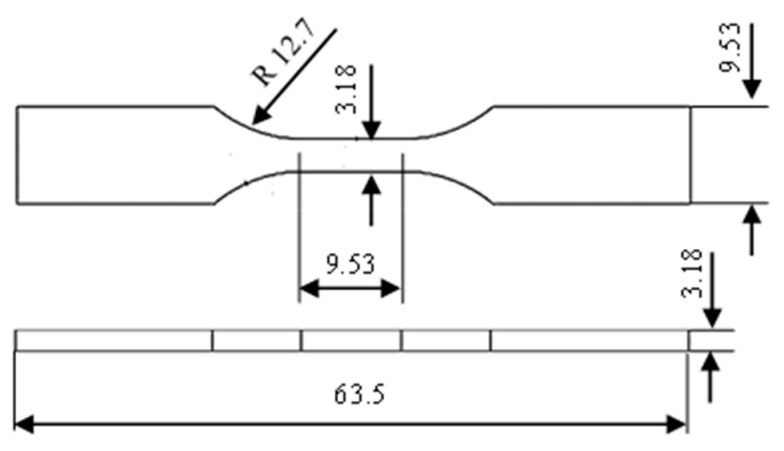
Sketch of a dog-bone sample according to ASTM D638 standard (Type V). All dimensions are in mm.

The tensile properties of 3D-printed material samples, as illustrated in Figure 3, including tensile strength at yield, tensile modulus and elongation at break, were determined using a Lloyd EZ50 (Lloyd Instruments Ltd., Bognor Regis, UK) universal testing machine (UTM). The tensile testing method and the associated sample type in this study were based on ASTM D638 standard. Tensile tests were conducted at room temperature with a crosshead speed of 1 mm/min and a load cell capacity of 10 kN.

The hardness data of 3D-printed samples were obtained using a digital shore D durometer ICHF-SHRD (Instrument Choice, Adelaide, Australia) based on ASTM D2240 standard [39]. Six measurements were taken at the grip sections for each sample by maintaining a distance at least 3 mm away from the sample edge, as well as between individual measurements.

Abrasion resistance tests were undertaken to determine abrasion loss by means of volume loss (mm^3^) using a DZ-323 rotary drum abrader according to ASTM D5963 standard. Here, volume loss was calculated from the ratio of mass loss over the density, where mass loss refers to the mass difference of a testing sample before and after the test. As such, volume loss can be corrected using the ratio *S*_0_*/S*, where *S*_0_ and *S* represent the nominal abrasiveness and actual abrasiveness of abrasive sheets in the test respectively. The density of material samples was determined with additional deionised (DI) water according to Archimedes’ principle [40]. The rest was carried out according to ASTM D5963 standard (method B) using a rotating test sample along with a standard rubber #1 as a reference material. Mass loss and density were calculated by weighing both the test sample and standard rubber before and after the test with a digital scale (accuracy: ±0.1 mg) equipped with a density kit (Mettler-Toledo Ltd., Melbourne, Australia). The densities of the test samples and standard rubber were determined using the following equation

(1) $$\rho =\frac{A}{A-B}\left(\rho_{0}-\rho_{L}\right)+\rho_{L}$$

where *ρ, ρ*_0_ and *ρ_L_* represent the density of the test sample, the DI density of 1 g·cm^−3^, and the air density of 0.0012 g·cm^−3^ respectively. *A* and *B* are the weights of the test sample in air and DI accordingly.

The abrasion loss of a test sample can be calculated as follows:

(2) $$A_{B}=\frac{∆m_{t}\times S_{0}}{d_{t}\times S}$$

where *A_B_,* Δ*m_t_* and *d_t_* denote the abrasion loss determined using method B, the mass loss, and the density of the test samples respectively. Furthermore, *S*_0_ and *S* are indicative of normal abrasiveness (i.e., *S*_0_ = 200 mg) and the abrasiveness of the standard rubber respectively. At the end of each abrasion resistance test, wear debris particles on the abrasive paper were removed using a brush. The test parameters are presented in Table S4 (Supporting Information).

### 2.4. Material Characterisation

A Fourier-transform infrared (FTIR) Nicolet iS50 spectrometer (Thermo Fisher, Waltham, MA, USA) was employed to evaluate chemical structures on the basis of the FTIR spectra of TPU, HNT, DMF, TPU/HNT/DMF mixture, and TPU/HNT nanocomposite films. It was conducted at room temperature using an attenuated total reflectance (ATR) technique. Meanwhile, sample spectra were recorded in a wavenumber range of 4000 to 400 cm^−1^ at a resolution of 4 cm^−1^.

Thermal stability behaviour of material samples was assessed by a thermogravimetric analyser SDT 2960 (TA Instruments, New Castle, DE, USA). In particular, the weight losses of neat TPU and TPU/HNT nanocomposites were investigated using a cryofill liquid nitrogen cooling system. Material samples about 20 mg were placed in the sample cup. Thermal stability was evaluated at a heating rate of 10 °C/min in a temperature range of 25–900 °C, as per recommendation by Adak et al. [34].

*T_g_*, crystallisation temperature (*T_c_*) and melting temperature (*T_m_*) of 3D-printed samples were evaluated using a differential scanning calorimeter Discovery DSC 25 (Texas Instruments, Dallas, TX, USA). Material samples about 4 mg were sealed in an aluminium pan and heated from −50 to 240 °C with a heating rate of 20 °C/min. They were then subjected to isothermal conditions at 240 °C for 5 min to remove any thermal history. Afterwards, the samples were cooled from 240 to −50 °C at a cooling rate of 20 °C/min. The same heating–cooling scan was repeated for a second time. *T_g_* and *T_m_* were determined from the first heating scan, while the effect of HNT inclusion in PU matrices on *T_c_* was investigated during the second scan after removing thermal history. The degree of crystallinity (*χ_c_*) was calculated using Equation (3) given below according to Deng et al. [41].

(3) $$\chi_{c}(\%)=\frac{\Delta H_{m}-{\Delta H}_{c}}{\Delta H_{m}^{0}}\times \frac{100}{1-w_{f}}$$

where ∆*H_m_* and ∆*H_c_* are the heat of fusion and the heat of the crystallisation of TPU/HNT nanocomposites respectively. Meanwhile, $\Delta H_{m}^{0}$ and *w_f_* represent 100% crystalline PU and the weight fraction of HNTs in TPU/HNT nanocomposites. $\Delta H_{m}^{0}$ is approximately 140 J·g^−1^ for neat PU, as mentioned previously by Cao et al. [42].

Surface morphology of 3D printed material samples after fracture in tensile tests was examined using scanning electron microscopy (SEM) via a Clara field emission–scanning electron microscope (FESEM) (Tescan GmbH, Dortmund, Germany). Selected material samples were cut, their fracture surface areas cleaned, and then mounted on SEM tubes (diameter: 12.5 mm) using double-sided carbon tape. Sputter coating was conducted on material samples using carbon layers (layer thickness: 20 nm) to achieve good electrical conductivity and avoid any charging effect for better image clarity.

On the other hand, HNT dispersion in TPU/HNT nanocomposites was examined by transmission electron microscopy (TEM). An FEI Talos FS200X G2 (Thermo Fisher Scientific, Waltham, MA, USA) transmission electron microscope was utilised with a field emission gun (TEG). Ultrathin TEM samples (average thickness: 100 nm) were sliced using an ultramicrotome Leica EM UC6 with a glass knife, and then collected on 300-mesh copper grids prior to TEM analysis.

## 3. Statistical Analysis

A Taguchi orthogonal array (OA) was employed to evaluate the mechanical properties of 3D-printed parts including tensile strength at yield, hardness, and abrasion resistance, along with their dimensional errors and surface roughness. This was based upon a mixed selection of input parameters such as HNT level at six levels in addition to nozzle temperature, print speed, infill density and layer height at three levels. As a result, L_18_ OA was applied with the assumption of minimal factorial interaction with DoEs in this study, in accordance with Table 3. At least three samples were prepared per material batch for each sample test.

DoE responses are referred to as maximising tensile properties and hardness, as well as minimising abrasion loss of printed parts by calculating the sum of signal-to-noise (*S/N*) ratios. The *S/N* ratio is an essential indicator in robust design and manufacturing for enhancing the quality and measurements while reducing variability [43]. The “larger-the-better” and “smaller-the-better” criteria [44] were implemented in DoEs using Equations (4) and (5) respectively. The “larger-the-better” criterion was used to evaluate significant parameters, with the aim of maximising the tensile properties and hardness of the samples, as opposed to the “smaller-the-better” criterion, which was employed with the aim of minimising abrasion loss.

(4) $$S/N_{i}=-10{Log}_{10}\frac{1}{n_{i}}\left(\sum_{u=1}^{n_{i}}\frac{1}{y_{u}^{2}}\right)$$

(5) $$S/N_{i}=-10{Log}_{10}\frac{1}{n_{i}}\left(\sum_{u=1}^{n_{i}}y_{u}^{2}\right)$$

The notations *i, u*, *n_i_* and *y_u_,* in Equations (4) and (5) represent the number of experiments, trial number, number of trials for the *i*th experiment, and data observed as output response. In general, mathematically higher *S/N* ratios yield better results, owing to their corresponding highest quality with the minimum variance, which is the fundamental criterion used to primarily select the optimal factor-level combination in this study.

## 4. Results and Discussion

### 4.1. Tensile Properties

**Figure 4:**
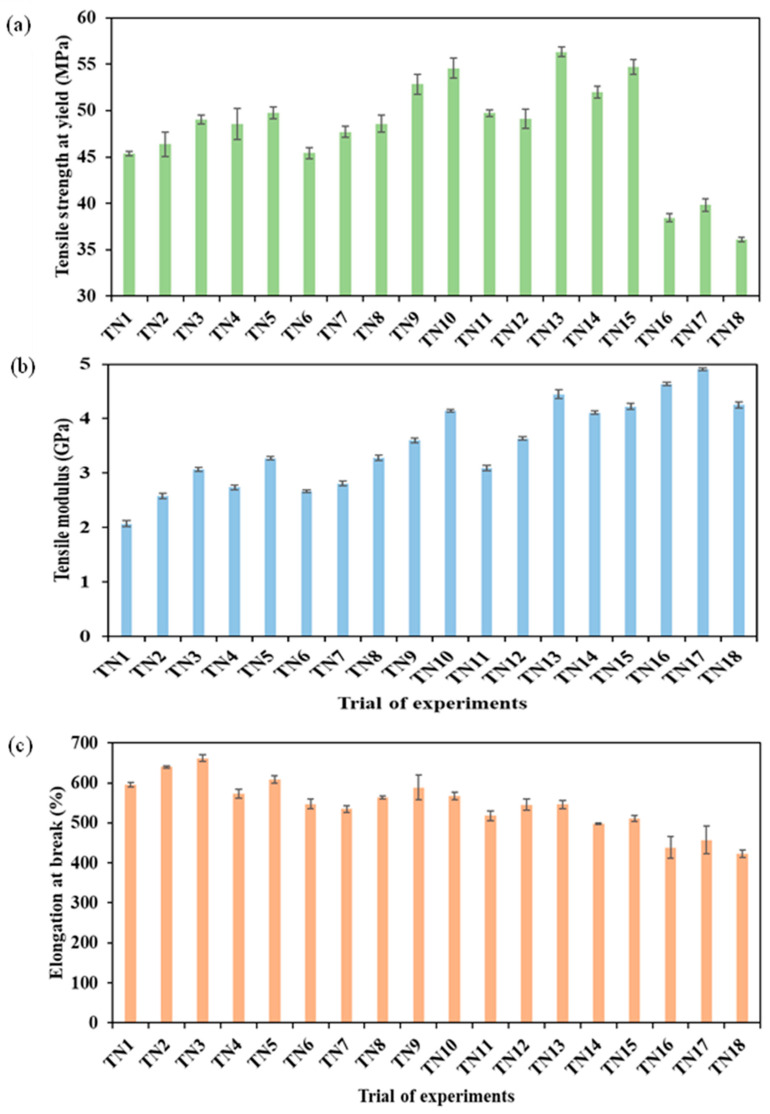
(**a**) Tensile strength at yield, (**b**) tensile modulus, and (**c**) elongation at break of dog-bone samples.

As can be seen from Figure 4a, the tensile strength of TPU/HNT nanocomposites was enhanced with HNT level of 8 wt%, with the highest tensile strength of 56.34 MPa being achieved for TN13, which was 24.2% higher than that of TN1 (i.e., neat TPU), at 45.35 MPa. This phenomenon suggests that good interfacial bonding occurs between HNTs and TPU matrices, which is in good agreement with Mahunaki et al. [25]. However, the tensile strength declined significantly when the HNT level was increased to 10 wt%, owing to the typical issue of HNT agglomeration. As such, the lowest tensile strength of 36.11 MPa was detected for TN18 due to weak filler–matrix interactions. On the other hand, the inclusion of HNTs as rigid nanofillers inevitably increased the tensile moduli of TPU/HNT nanocomposites [45], as illustrated in Figure 4b. The maximum tensile modulus of 4.91 GPa was achieved for TN17 with the addition of 10 wt% HNTs, in contrast to the minimum modulus of 2.07 GPa for TN1. In general, composite materials can benefit from the inclusion of rigid fillers to enhance their stiffness, as is the case for TPU/HNT nanocomposite as well. Rigid HNTs greatly restrict the chain mobility of TPU molecules, resulting in a reduction in material flexibility and an increase in stiffness, which is similar to what has been reported in previous work by Sulong et al. [46]. As such, elongation at break for dog-bone samples decreased with increasing HNT level, Figure 4c. It is well understood that a higher level of rigid nanofillers like HNTs makes it possible to alter the material characteristics in order to obtain a more brittle nature. In particular, the lowest elongation at break was 422.49% for TN18 with the inclusion of 10 wt%.

**Figure 5:**
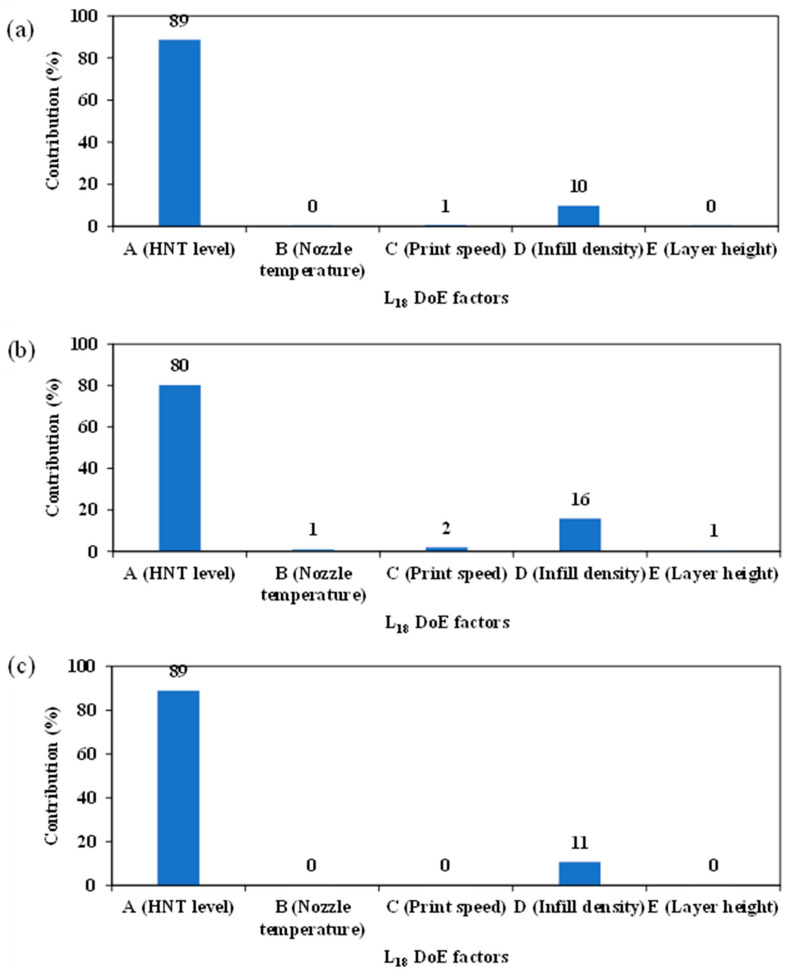
Pareto ANOVA for enhancing tensile properties of TPU/HNT nanocomposites: (**a**) tensile strength at yield, (**b**) tensile modulus and (**c**) elongation at break for dog-bone samples.

Pareto ANOVA was conducted with respect to the tensile properties of TPU/HNT nanocomposites. Significant factors were determined according to the criterion that cumulative contribution percentage should be over 90% [43], along with corresponding ANOVA diagrams exhibited in Figure 5. As can be seen from Figure 5a, HNT level (factor A) and infill density (factor D) are categorised as significant factors, with a cumulative contribution percentage of approximately 99%. It is evidently shown that the inclusion of nanofillers and air gaps between the printed layers can adversely influence the tensile strength of TPU/HNT nanocomposites, as reported elsewhere [47]. Meanwhile, nozzle temperature (factor B), print speed (factor C) and layer height (factor E) can be deemed as non-significant factors. However, Vidakis et al. [48] reported that increasing printing temperature and layer height had negative effect on tensile strength instead. Similarly, HNT level and infill density also induce dominant effect on tensile modulus of TPU/HNT nanocomposites, leading to a cumulative contribution of approximately 96%, as illustrated in Figure 5b. Similar to tensile strength at yield and tensile modulus, HNT level and infill density also significantly affect elongation at break with a total contribution of 100%, as illustrated in Figure 5c. Print speed and layer thickness tend to be non-significant processing parameters with respect to elongation at break for dog-bone samples, in contrast to previous results reported by Kandi et al. [49], where it was suggested that elongation of 3D-printed samples declined at the higher print speed and larger layer thickness.

**Figure 6:**
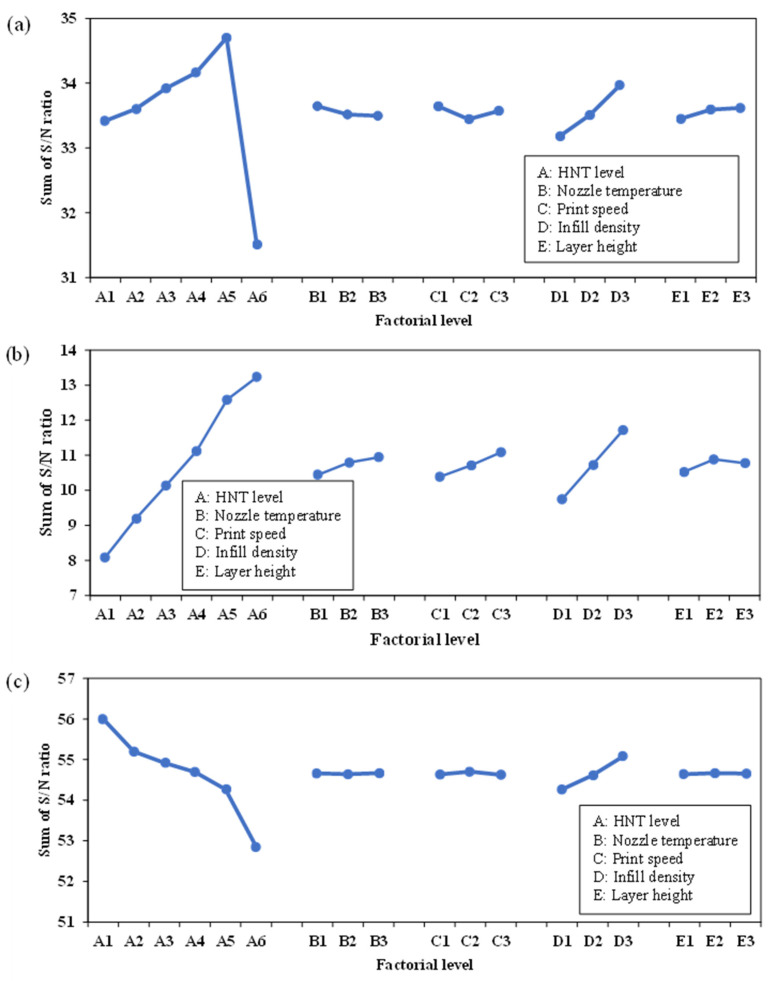
Sum of *S/N* ratios at different factorial levels for improving the tensile properties of TPU/HNT nanocomposites: (**a**) tensile strength at yield, (**b**) tensile modulus and (**c**) elongation at break for dog-bone samples.

The “larger-the-better” criterion is employed in Taguchi DoE analysis in this study to identify the optimum factor-level combination for maximising mechanical properties. In general, mathematically the greater the sum of *S/N* ratios, the better the response in terms of factorial effect. The better response in terms of factorial effect is indicated by the greater sum of *S/N* ratios. Overall, HNT level (factor A) is prevalent in impacting tensile strength, as shown in Figure 6a, where increasing the HNT level to 8 wt% enhances the sum of *S/N* ratios, followed by a sharply declining trend at an HNT level of 10 wt%, which can be attributed to localised HNT agglomeration in nanocomposite samples. Such agglomeration effect may cause stress concentration zones due to embedded undispersed HNTs that are prone to crack failure, as well as the creation of weak filler–matrix interfacial bonding, thus resulting in a sharp decline in the tensile strength of dog-bone nanocomposite samples. Infill density was determined to be the second significant factor. The maximum tensile strength was achieved at the highest infill density of 100%, as expected, which is in good agreement with Wang et al. [50]. Meanwhile, other factors, including nozzle temperature, print speed and layer height, had minor effects on tensile strength. The optimum factor-level combination with the aim of achieving maximum tensile strength was obtained when HNT level, nozzle temperature, print speed, infill density and layer height were 8 wt%, 210 °C, 10 mm·s^−1^, 100% and 0.4 mm respectively (i.e., A_5_B_1_C_1_D_3_E_3_).

As can be observed in Figure 6b, the sum of *S/N* ratios increased monotonically with the addition of HNTs up to 10 wt% as rigid nanofillers with the aim of enhancing the stiffness of TPU/HNT nanocomposites. The optimum factor-level combination associated with the highest sum of *S/N* ratios in response to maximum tensile modulus is comprised of HNT level of 10 wt%, nozzle temperature of 230 °C, print speed of 30 mm·s^−1^, infill density of 100% and layer height of 0.3 mm (i.e., A_6_B_3_C_3_D_3_E_2_). Conversely, there appeared to be a reverse trend with respect to elongation at break, especially for HNT level (factor A), as shown in Figure 6c. This finding can be ascribed to the brittle character and nanofiller agglomeration of embedded rigid HNTs in TPU/HNT nanocomposites. In relation to elongation at break, Le et al. [51] reported quite different results, indicating that the maximum elongation at break could be achieved at the lowest level of infill density. The corresponding optimum factor-level combination for maximum elongation at break consists of an HNT level of 0 wt%, a nozzle temperature of 230 °C, a print speed of 20 mm·s^−1^, an infill density of 100% and a layer height of 0.3 mm (i.e., A_1_B_3_C_2_D_3_E_2_).

### 4.2. Hardness and Abrasion Resistance

**Figure 7:**
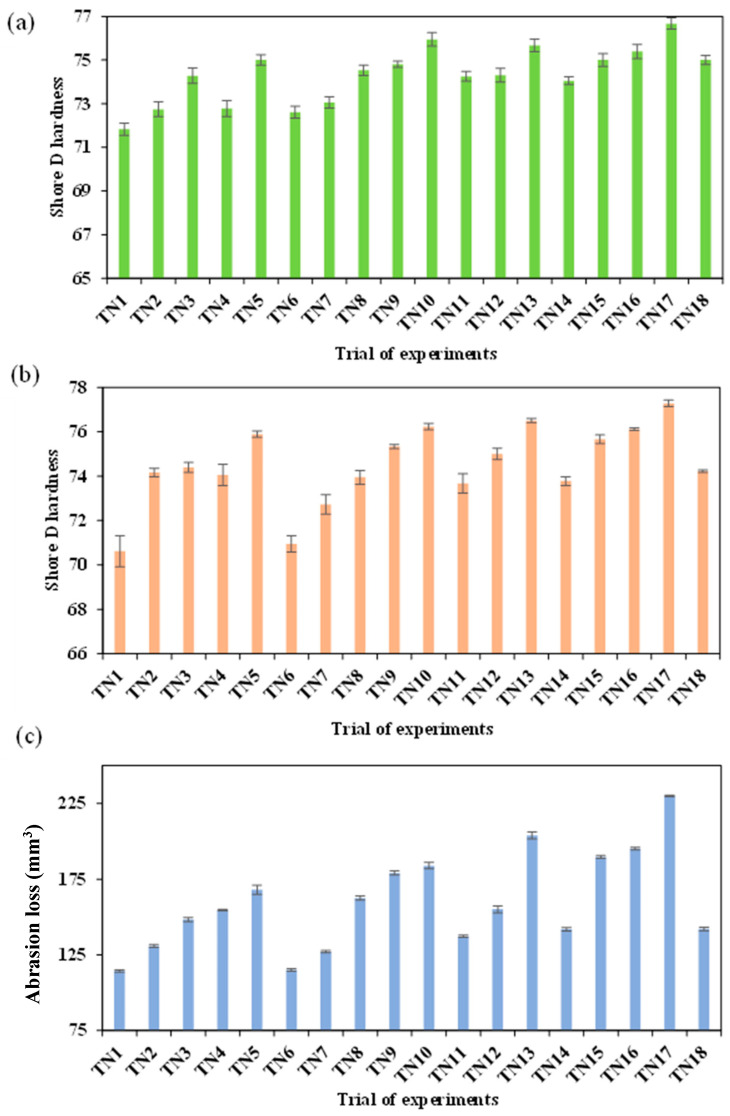
(**a**) Hardness of dog-bone samples; (**b**) hardness and (**c**) abrasion resistance of cylindrical samples.

Hardness and abrasion resistance results are reported in Figure 7 for the DoE study. It can be observed that increasing the HNT level improved the Shore D hardness of TPU/HNT nanocomposite dog-bone samples from 71.50 (TN1) to 76.67 (TN17), as shown in Figure 7a, which could be related to embedded HNTs to obstruct hydrogen bonding between polymeric segments with the formation of new hydrogen bonds between HNTs and TPU molecular chains [25]. It has been proven that the inherent hardness and stiffness of nanofillers makes it possible to enhance the hardness of corresponding nanocomposites, as reported by Mohamed et al. [52] when preparing TPU/2 wt% HNT composites for corrosive coating application.

Shore D hardness data of cylindrical samples are similar to those of their dog-bone counterparts, as depicted in Figure 7b. The lowest and highest hardness values were determined to be 70.61 for TN1 and 77.28 for TN17. The fundamental principle is based on volume loss to represent abrasion resistance for harder materials [53], which is also the case for the cylindrical samples used in this study according to Figure 7c. As such, TPU/HNT nanocomposites at higher HNT levels inevitably exhibit greater volume loss, resulting in a reduction in their abrasion resistance. This finding is in good accordance with that reported by Mohamed et al. [52], where the inclusion of 2 wt% HNTs enhanced the abrasion loss by 96%, as opposed to that of neat TPU.

**Figure 8:**
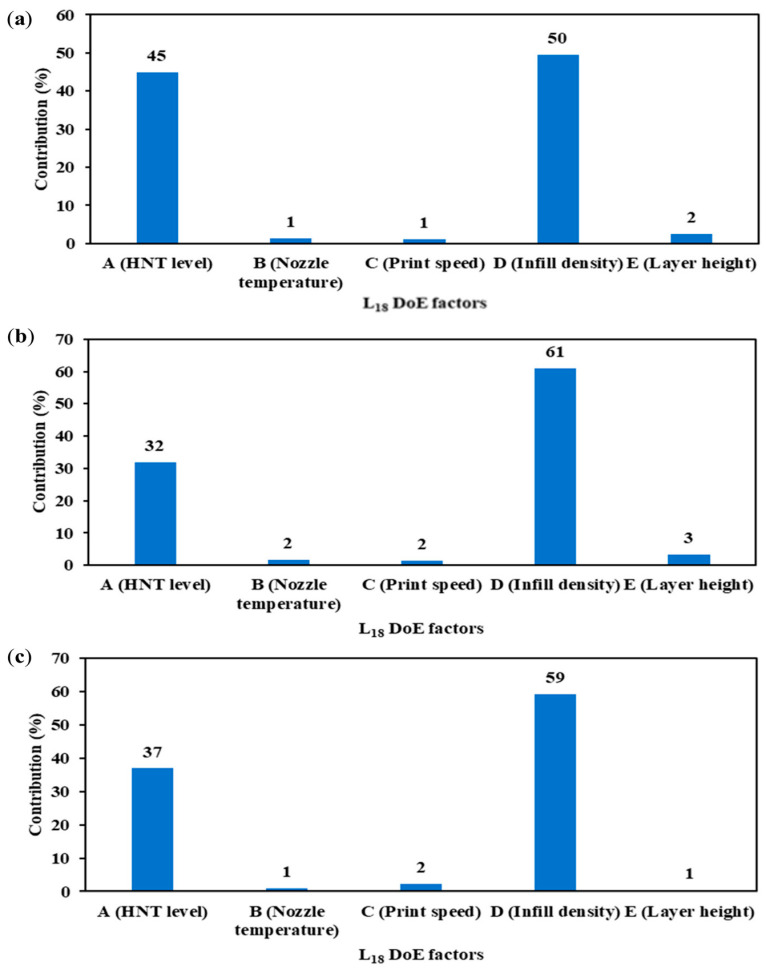
Pareto ANOVA diagrams for maximising (**a**) the hardness of dog-bone samples, as well as (**b**) the hardness and (**c**) abrasion resistance of cylindrical samples.

Figure 8a demonstrates that infill density (factor D) and HNT level (factor A) were determined to be the two significant factors, with contribution percentages of 50 and 45% respectively in terms of the hardness of dog-bone samples. The other non-significant factors involving nozzle temperature (factor B), print speed (factor C) and layer height (factor E) make only marginal contributions of 1, 1 and 2% accordingly. In comparison, the hardness of the cylindrical samples is also significantly influenced by infill density and HNT level with a cumulative contribution percentage of 93% shown in Figure 8b. However, nozzle temperature, print speed and layer height make only slight contributions of 2, 2 and 3% respectively. As for the abrasion resistance of cylindrical samples, infill density and HNT level induce the highest contributions of 59 and 37%, despite the minimal effect of other factors, as shown in Figure 8c.

**Figure 9:**
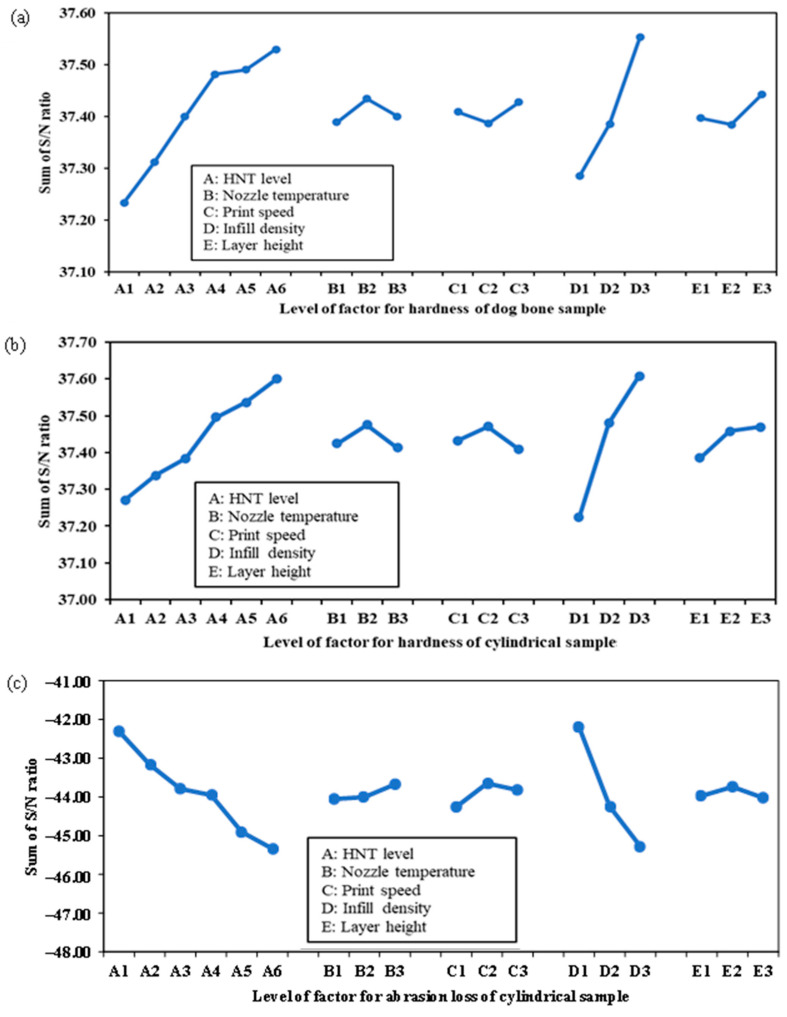
Sum of *S/N* ratio at different factorial levels for (**a**) hardness of dog-bone samples, as well as (**b**) hardness and (**c**) abrasion loss of cylindrical samples.

The optimum factor-level combination in response to the maximum hardness is based on the “larger-the-better” criterion in the Taguchi DoEs for both dog-bone and cylindrical samples. Figure 9a indicates that increasing infill density increases the sum of *S/N* ratios for hardness, since it yields less air gap between printed layers, leading to denser materials and better deformation resistance [54]. The addition of HNTs induces more compact and stronger hydrogen bonds between HNT nanofillers and TPU molecular chains. The optimum factor-level combination, resulting from the highest sum of *S/N* ratios in response to the maximum hardness of the dog-bone samples, is with reference to an HNT level of 10 wt%, a nozzle temperature of 220 °C, a print speed of 30 mm·s^−1^, an infill density of 100% and a layer thickness of 0.4 mm (i.e., A_6_B_2_C_3_D_3_E_3_).

A similar trend is evidently shown for the cylindrical samples, except for print speed (factor C) and layer height (factor E), as illustrated in Figure 9b. The optimum factor-level combination for cylindrical samples was achieved at an HNT level of 10 wt%, nozzle temperature of 220 °C, print speed of 20 mm·s^−1^, infill density of 100%, and layer height of 0.4 mm, namely, A_6_B_2_C_2_D_3_E_3_. As mentioned earlier, abrasion resistance is characterised by the resistance of materials to wear deformation, and generally better performance in terms of abrasion durability is represented by smaller abrasion loss [55]. Hence, a “smaller-the-better” criterion was used to determine the optimum factor-level combination in terms of achieving minimum abrasion loss in the cylindrical samples of TPU/HNT nanocomposites. It was found that lower infill density and HNT level result in better abrasion resistance, which is an opposite tendency to that of hardness, as shown in Figure 9c. It can be observed that the highest abrasion resistance was obtained based on the optimum factor-level combination, comprising an HNT level of 0 wt%, a nozzle temperature of 230 °C, a print speed of 20 mm·s^−1^, an infill density of 40% and a layer height of 0.3 mm (i.e., A_1_B_3_C_2_D_1_E_2_).

**Figure 10:**
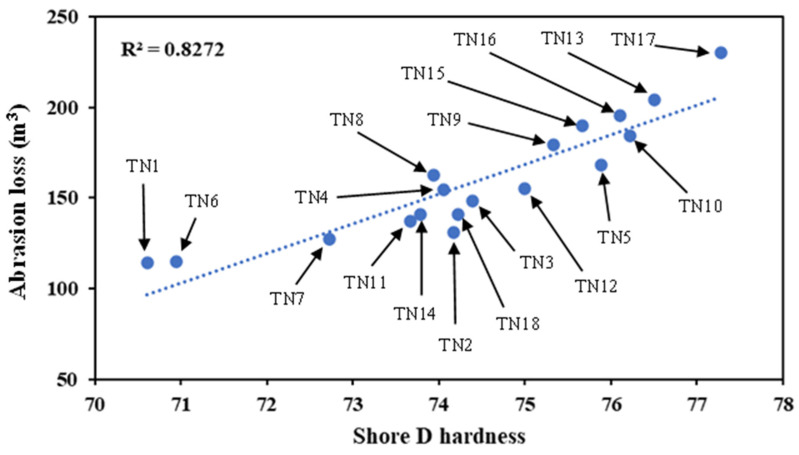
Linear relationship between hardness and abrasion loss in TPU/HNT nanocomposite cylindrical samples.

It was previously stated that the inclusion of HNTs in TPU matrices could enhance the hardness of corresponding nanocomposites, and higher hardness leads to more severe volume loss. As seen from Figure 10, abrasion loss becomes more pronounced with increasing Shore D hardness in a monotonically increasing manner, as evidenced by R-square (R²) values of up to approximately 0.83 based on the linear regression.

Table 4 summaries optimal factor-level combinations for enhanced mechanical properties of TPU/HNT nanocomposites. In tensile, hardness and abrasion tests, TN13, TN3 and TN1 possessed the maximum tensile strength, elongation at break and abrasive resistance respectively in addition to TN17, which possessed the highest tensile modulus and hardness. This study was mainly focused on tensile strength and abrasion resistance with the comparison of corresponding compressive properties of TPU/HNT nanocomposites, as opposed to elongation at break. As typical cases, TN13, TN17 and TN1 were selected for material characterisation using SEM and TEM to evaluate their morphological structures and HNT nanofiller dispersion in the nanocomposite systems, along with the performance of FTIR spectroscopy, thermogravimetric analysis (TGA) and differential scanning calorimetry (DSC) to investigate their corresponding chemical structures and thermal properties.

### 4.3. Morphology of TPU/HNT Nanocomposites

**Figure 11:**
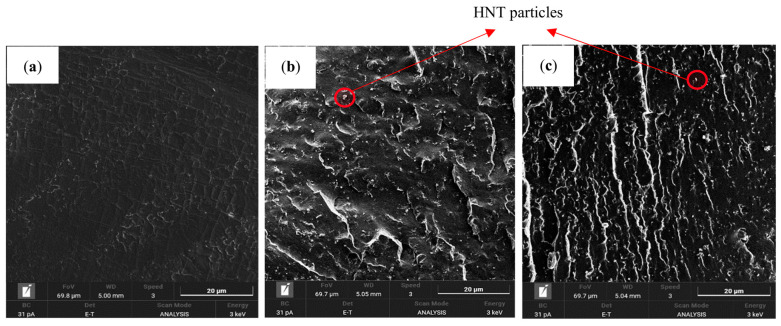
SEM micrographs: (**a**) neat TPU (TN1); TPU/HNT nanocomposites reinforced with (**b**) 8 wt% HNTs (TN13) and (**c**) 10 wt% HNTs (TN17).

Figure 11 presents the SEM micrographs of neat TPU (TN1) and TPU/HNT nanocomposites with the inclusion of 8 wt% HNTs (TN13) and 10 wt% HNTs (TN17). It can be observed that TN13 possesses better nanofiller dispersion compared to TN17, as illustrated in Figure 11b,c. No apparent porous structures can be observed for either TPU in Figure 11a or its corresponding nanocomposites, which is a clear sign that strong filler–matrix interfacial bonding has taken place [25].

**Figure 12:**
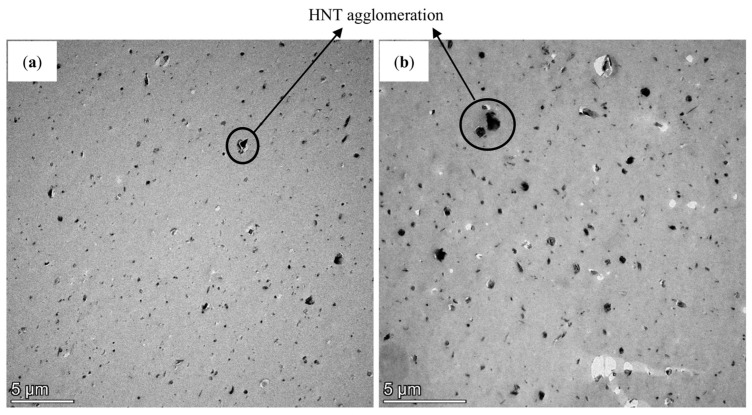
TEM micrographs: (**a**) TPU/HNT nanocomposites reinforced with 8 wt% HNTs (TN13) and (**b**) 10 wt% HNTs (TN17).

On the other hand, in the TEM micrographs presented in Figure 12, the darker regions represent HNT nanofillers while the lighter areas are referred to as TPU matrices. As for nanocomposites reinforced with 8 wt% HNTs (TN13), HNTs were detected to be relatively well dispersed within the matrices in a random orientation, despite a certain degree of localised HNT agglomeration, as shown in Figure 12a. When HNT level reaches 10 wt% for TN17, HNT aggregation becomes more pronounced, as can be observed in Figure 12b. However, such HNT aggregates in a size of 1–2 µm are just dotted around reasonably dispersed HNTs, and thus do not result in severe HNT agglomeration. Non-homogeneous HNT dispersion with typical agglomeration is detrimental to achieving a smooth 3D printing process, with the occurrence of material clogging and severe HNT agglomeration, thus inducing the blockage of nozzle tip of the 3D printer. In particular, with respect to the FDM technique, this phenomenon may result in a deterioration of the extrudability of hot filaments and material deposition onto the build platform.

### 4.4. FTIR Analysis

**Figure 13:**
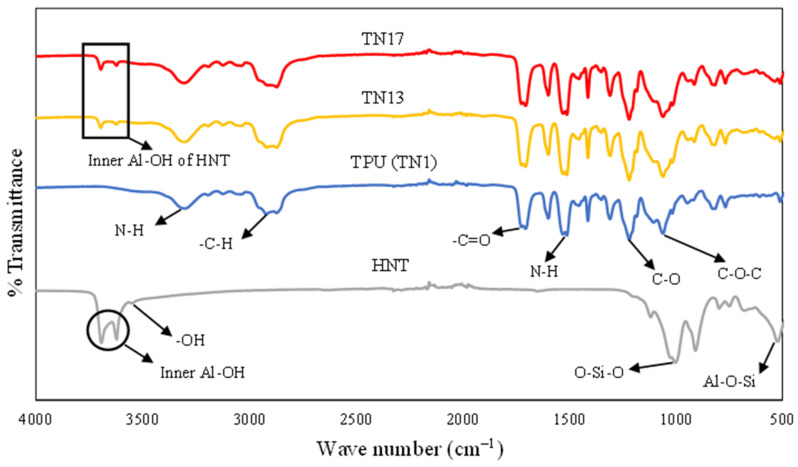
FTIR spectra of HNT, TPU (TN1) and TPU/HNT nanocomposites (TN13 and TN17).

Figure 13 presents the FTIR spectra of HNTs, neat TPU (TN1) and TPU/HNT nanocomposites (TN13 and TN17). The FTIR peaks appearing at 3690 and 3620 cm^−1^ for HNTs can be attributed to the stretching vibrations of inner surface hydroxyl groups (Al-OH). Additionally, the absorption band occurring at 3542 cm^−1^ corresponds to O-H stretching with respect to water [56]. On the other hand, related bands at 998 and 523 cm^−1^ arise from the stretching vibration of O-Si-O, as well as the deformation vibration of Al-O-Si [57,58]. Finally, it appears that the FTIR peaks assigned to 909 and 457 cm^−1^ are due to the deformation vibration of inner hydroxyl groups.

Furthermore, the absorption band at 3292 cm^−1^ corresponds to the NH bond stretching of TPU [59], while the peak detected at 2910 cm^−1^ is associated with its –CH_2_ stretching [60]. The band obtained at 1704 cm^−1^ is assigned to hydrogen bonds between the N–H and C=O groups in the hard segments and the ester groups for urethane linkage in the soft segments [61]. The other characteristic bands at 1510, 1216 and 1057 cm^−1^ can be attributed to N-H bending vibration, C–O stretching vibration and ester C–O–C symmetric stretching vibration respectively [59]. Even though TN13 and TN17 have different filler levels (i.e., 8 and 10 wt% HNTs), and were printed using different 3D printing settings (i.e., nozzle temperature, print speed, infill density and layer height), the FTIR spectra of both samples are very similar. This may suggest that different HNT levels and printing settings may have minor impacts on the chemical structures of TPU/HNT nanocomposites.

### 4.5. Thermal Stability

**Figure 14:**
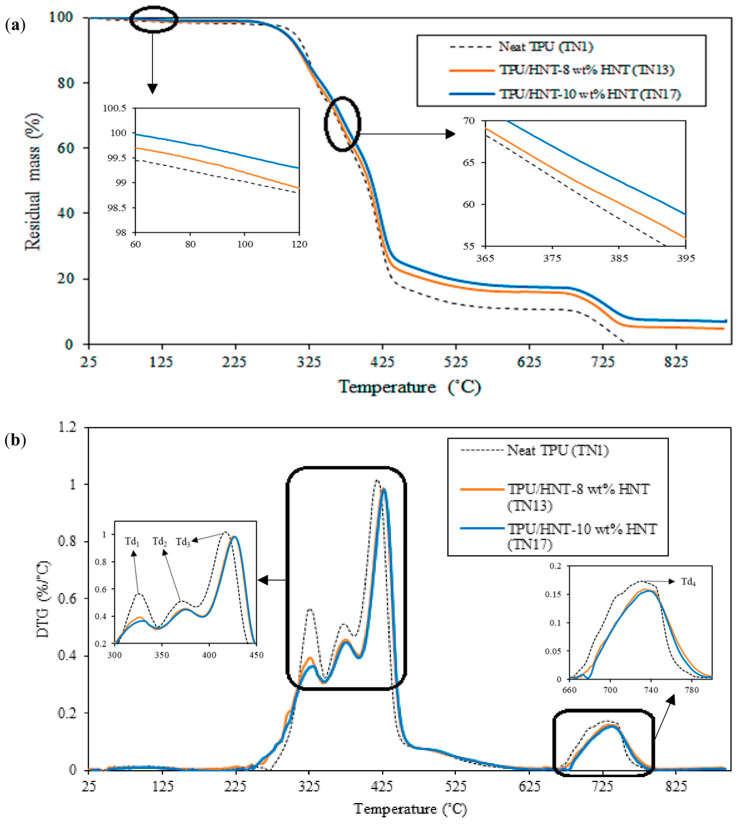
TGA thermograms of TPU (TN1) and TPU/HNT nanocomposites (TN13 and TN17): (**a**) TGA curves and (**b**) DTG curves. *T_d_* represents decomposition temperature.

The thermal stability of 3D-printed TPU and TPU/HNT nanocomposites was characterised using TGA, as depicted in Figure 14. It can be observed that the maximum degradation was detected on the basis of four degradation peaks in the derivative thermogravimetric (DTG) spectra, which are represented by four decomposition temperatures, namely, *T_d_*_1_, *T_d_*_2_, *T_d_*_3_ and *T_d_*_4_ respectively in Table 5.

The first peak of residual mass in the TGA curve for neat TPU (TN1) in the temperature range 239–346 °C is attributed to the release of water molecular bonds in empty tubular lumens of HNTs [25]. It is followed by the second peak, taking place between 346 and 386 °C, which is associated with the dissociation of urethane, as the hard segments break down into alcohol and isocyanate [50]. Delebecq et al. [62] reported that the depolymerisation reaction of TPU was reversible, and equilibrium was attained almost instantaneously. Another study by Lee et al. [63] confirmed that urethane groups were constructed through the reaction between phenolic hydroxyl groups and isocyanates, which is generally known to be reversible at high temperatures. It is suggested that a reversible behaviour occurs for the dissociation of PU bonds. Meanwhile, the third peak, in accordance with the breakage of soft segments of polyether polyol [64], is assigned to the temperature range of 386–458 °C, while the degradation of carbon occurs at temperatures between 662–783 °C.

As for TN13, the four sequential peaks are identified at the temperature ranges of 240–348 °C, 348–393 °C, 393–471 °C and 670–802 °C respectively. In contrast, the mass loss of TN17 occurs at temperature levels between 242–348 °C for the first peak, 348–393 °C for the second peak, 393–472 °C for the third peak, and 678–790 °C for the last peak. As shown in Table 5, the inclusion of HNTs appears to enhance the thermal stability of TPU/HNT nanocomposites, especially for the third peak in the range 416–426 °C for TN13 and approximately between 416–427 °C for TN17. TN17 with the addition of 10 wt% HNTs possesses a much higher maximum decomposition temperature than TN13 reinforced with 8 wt% HNTs, which suggests that increasing HNT level can improve the thermal stability of TPU.

### 4.6. DSC Measurements

**Figure 15:**
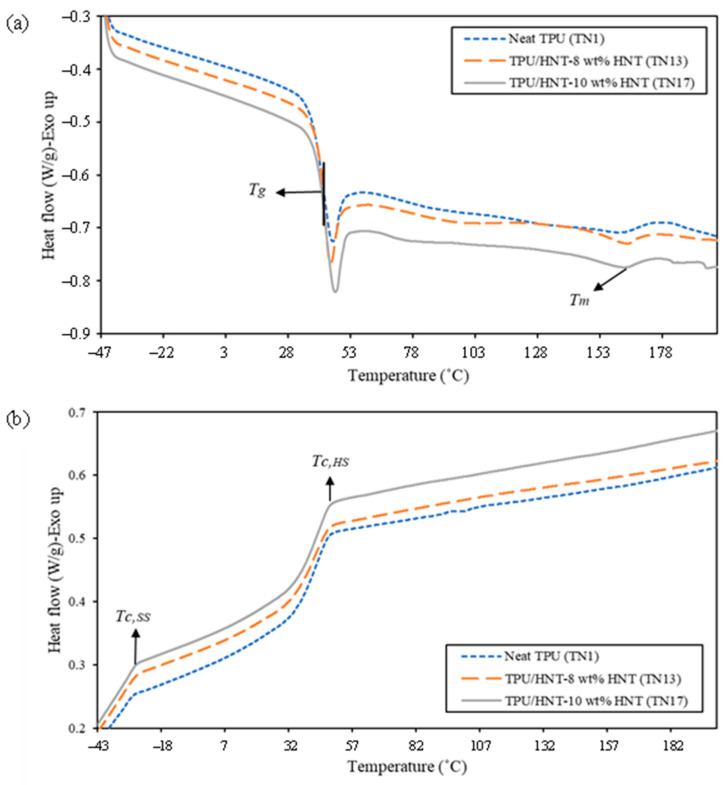
DSC thermograms of TPU and TPU/HNT nanocomposites: (**a**) heating scan and (**b**) cooling scan.

Figure 15 depicts the DSC thermograms of neat TPU (TN1) and TPU/HNT nanocomposites (TN13 and TN17), along with associated thermal parameters listed in Table 6. It is well understood that TPU is comprised of hard and soft segments, where *T_g_* of hard segments, in connection with the covalent bonds of NCO and OH groups, appears to influence the mobility of polymeric chains [65]. The presence of HNTs in this study slightly promotes *T_g_* from 40.97 °C for neat TPU (TN1) to 41.56 and 42.36 °C for TPU/HNT nanocomposites (i.e., TN13 and TN17), owing to well-dispersed rigid HNTs, which restrict the chain mobility of TPU matrices in corresponding nanocomposite systems [66].

As expected, the addition of HNTs in TPU matrices yielded typical phase separation, thus resulting in higher melting peaks from 161.31 °C (TN1) to 164.01 °C (TN13), as shown in Figure 15a and Table 6. The phase separation of TPU/HNT nanocomposites is attributed to HNT absorption on hard segments. When the HNT level reaches 10 wt%, the extraction of hard segments from the mixture tends to increase the purification of the soft phase [67], causing a shift of the melting peak to a relatively low temperature of 163.03 °C.

The inclusion of HNTs to polymer matrices leads to a higher degree of crystallinity owing to the nucleating effect of HNT nanofillers [66]. As seen in Table 6, TPU/HNT nanocomposites with an HNT level of 10 wt% have an *Χ_c_* of 4.78%, when compared with neat TPU with an *Χ_c_* of 2.84%. Additionally, the DSC peak associated with the crystallisation temperature of hard segments (*T_c,HS_*) was observed to shift from 47.65 °C for neat TPU (TN1) to 48.42 and 49.43 °C for TPU/HNT nanocomposites when reinforced with 8 wt% (TN13) and 10 wt% HNTs (TN17) respectively, while the crystallisation temperature for the soft segments (*T_c,SS_*) shifts from −31.18 °C (TN1) to −29.94 °C (TN13) and −27.87 °C (TN17) respectively, as shown in Figure 15b and Table 5. It is noteworthy that higher *T_c_* reveals an increasing tendency towards phase separation, as mentioned earlier by Mamaqani et al. [68].

## 5. Conclusions

This study investigated the effects of HNT level in a range of 2–10 wt%, as well as 3D printing parameters including nozzle temperature, print speed, infill density and layer height, on the mechanical properties of TPU/HNT nanocomposites. Taguchi DoEs were proven to be effective for determining the optimum factor-level combinations for individual output responses with respect to mechanical properties. These results suggest that HNT level and infill density can significantly impact the tensile properties and hardness of dog-bone samples, in a similar case for the response to hardness and abrasion resistance of cylindrical samples.

Overall, the maximum tensile strength of TPU/HNT nanocomposites of 56.34 MPa was obtained with the addition of 8 wt% HNTs. Furthermore, the inclusion of 10 wt% HNTs (TN17) induced the highest tensile modulus and Shore D hardness of 4.91 GPa and 76.57 respectively. In particular, the most significant elongation at break of 661.77% was achieved for neat TPU (TN3). Meanwhile, the maximum Shore D hardness of 77.28 was reached with an HNT level of 10 wt% (TN17). Finally, the highest abrasion resistance, as represented by the minimum abrasion loss of 114.37 mm^3^, was obtained with neat TPU (TN1) as well.

In typical cases, both SEM and TEM micrographs demonstrate clearer signs of HNT agglomeration with the addition of 10 wt% HNTs for TN17, as opposed to TN13 and TN1. The increase in HNT level is supposed to enhance the thermal stability, *T_g_*, *T_c_*, *T_m_* and *Χ_c_* of 3D-printed TPU/HNT nanocomposites. Based on the subsequent material characterisation, TPU/HNT nanocomposites with an HNT level of 8 wt% (TN13) were proven to be a potential material candidate for achieving excellent tensile strength, thermal stability, and phase behaviour, with resulting multifunctional properties relevant to potential applications in piezoresistive sensors, scaffolds and protective gear.

## Figures and Tables

**Table 1:** Material formulation and 3D printing parameters.

Factor	Level					
HNT level (wt%)	0	2	4	6	8	10
Nozzle temperature (°C)	210		220		230	
Print speed (mm/s)	10		20		30	
Infill density (%)	40		70		100	
Layer height (mm)	0.2		0.3		0.4	

**Table 2:** Fixed 3D printing parameters.

Parameter	Specific Parameter	Setting
Quality	Shell thickness (mm)	1.6
	Initial layer thickness (mm)	0.5
	Initial layer line width (%)	120
	Top surface quality	precise
Fill	Bottom/top thickness (mm)	1.2
	Infill interface density	dense
	Infill type	triangle
	Infill overlap (%)	15
Temperature	Bed temperature (°C)	55
Speed	Travel speed (mm·s^−1^)	150
	Bottom layer speed (mm·s^−1^)	15
	Infill speed (mm/s)	30
Filament	Flow (%)	115
Retraction	Speed (mm/s)	30
	Distance (mm)	5
	Minimum travel (mm)	1.5
	Minimal extrusion before retracting (mm)	0.005

**Table 3:** L_18_ OA for TPU/HNT nanocomposite samples.

Exp.	Symbol	Factor				
		A (HNT Level wt%)	B (Nozzle Temperature °C)	C (Print Speed mm·s^−1^)	D (Infill Density %)	E (Layer Height mm)
1	TN1	0	210	10	40	0.2
2	TN2	0	220	20	70	0.3
3	TN3	0	230	30	100	0.4
4	TN4	2	210	10	70	0.3
5	TN5	2	220	20	100	0.4
6	TN6	2	230	30	40	0.2
7	TN7	4	210	20	40	0.4
8	TN8	4	220	30	70	0.2
9	TN9	4	230	10	100	0.3
10	TN10	6	210	30	100	0.3
11	TN11	6	220	10	40	0.4
12	TN12	6	230	20	70	0.2
13	TN13	8	210	20	100	0.2
14	TN14	8	220	30	40	0.3
15	TN15	8	230	10	70	0.4
16	TN16	10	210	30	70	0.4
17	TN17	10	220	10	100	0.2
18	TN18	10	230	20	40	0.3

**Table 4:** Summary of optimum factor-level combinations for enhanced mechanical properties of TPU/HNT nanocomposites.

Larger-the-Better L_18_ DoE Response	Significant Factor	Optimum Combination	Factor-Level Combination	Confirmation Test
Tensile strength at yield (MPa)	HNT level Infill density	A_5_B_1_C_1_D_3_E_3_	HNT level (wt%): 8 Nozzle temperature (°C): 210 Print speed (mm·s^−1^): 10 Infill density (%): 100 Layer height (mm): 0.4	57.293
Tensile modulus (MPa)	HNT level Infill density	A_6_B_3_C_3_D_3_E_2_	HNT level (wt%): 10 Nozzle temperature (°C): 230 Print speed (mm·s^−1^): 30 Infill density (%): 100 Layer height (mm): 0.3	5.003
Elongation at break (%)	HNT level Infill density	A_1_B_3_C_2_D_3_E_2_	HNT level (wt%): 0 Nozzle temperature (°C): 230 Print speed (mm·s^−1^): 20 Infill density (%): 100 Layer height (mm): 0.3	668.68
Shore D hardness (dog-bone samples)	Infill density HNT level	A_6_B_2_C_3_D_3_E_3_	HNT level (wt%): 10 Nozzle temperature (°C): 220 Print speed (mm·s^−1^): 30 Infill density (%): 100 Layer height (mm): 0.4	76.867
Shore D hardness (cylindrical samples)	Infill density HNT level	A_6_B_2_C_2_D_3_E_3_	HNT level (wt%): 10 Nozzle temperature (°C): 220 Print speed (mm·s^−1^): 20 Infill density (%): 100 Layer height (mm): 0.4	77.880
Smaller-the-better L_18_ DoE response	Significant factor	Optimum combination	Factor-level combination	Confirmation test
Abrasion loss (mm^3^)	Infill density HNT level	A_1_B_3_C_2_D_1_E_2_	HNT level (wt%): 0 Nozzle temperature (°C): 230 Print speed (mm·s^−1^): 20 Infill density (%): 40 Layer height (mm): 0.3	112.813

**Table 5:** TGA peak of TPU (TN1) and TPU/HNT nanocomposites (TN13 and TN17).

Sample Code	*T_d1_* (°C)	DTG (%/°C)	*T_d2_* (°C)	DTG (%/°C)	*T_d3_* (°C)	DTG (%/°C)	*T_d4_* (°C)	DTG (%/°C)
TN1	325.29	0.5659	371.16	0.5116	416.82	1.019	730.06	0.1729
TN13	326.1	0.393	376.11	0.4568	426.36	0.9878	732.88	0.1586
TN17	330.18	0.3655	375.4	0.4496	427.3	0.9843	737.5	0.1563

**Table 6:** DSC thermal properties of TPU and TPU/HNT nanocomposites.

Sample Code	*T_g_* (°C)	*T_c,SS_* (°C)	*T_c,HS_* (°C)	*T_m_* (°C)	∆*H_m_* (J/g)	∆*H_c_* (J/g)	*Χ_c_* (%)
TN1	40.97	−31.18	47.65	161.31	4.37	0.39	2.84
TN13	41.56	−29.94	48.42	164.01	6.41	0.46	4.62
TN17	42.36	−27.87	49.43	163.03	6.54	0.44	4.84

## Data Availability

The data presented in this study are available upon request from the corresponding author.

## Supplementary Materials

The following supporting information can be downloaded at: https://www.mdpi.com/article/10.3390/nano13131975/s1, Table S1: Physical and mechanical properties of TPU (MM-4520 grade) [27]; Table S2: Properties of HNTs [28]; Table S3: Data summary of filament extrusion parameters; Table S4: Data summary of abrasion resistance parameters.
