# Supplementary material for: Co-Influence of Nanofiller Content and 3D Printing Parameters on Mechanical Properties of Thermoplastic Polyurethane (TPU)/Halloysite Nanotube (HNT) Nanocomposites
Source: Nanomaterials (Basel). 2023 Jun 29;13(13):1975. doi: 10.3390/nano13131975 (PMC10343749; doi:10.3390/nano13131975)
Supplement: Supplementary file 1 [file nanomaterials-13-01975-s001.zip › nanomaterials-2447753-supplementary.pdf]

SUPPLEMENTARY INFORMATION

Wendy Triadji Nugroho [1], Yu Dong [1,*], Alokesh Pramanik [1], Zhixiao Zhang [2] and Seeram Ramakrishna [3]

[1] School of Civil and Mechanical Engineering, Curtin University, P.O. Box U1987, Perth, WA 6845, Australia; w.nugroho1@postgrad.curtin.edu.au (W.T.N.); alokesh.pramanik@curtin.edu.au (A.P.)
[2] School of Materials Science and Engineering, Hebei University of Engineering, Handan 056038, China; zhixiao351@hebeu.edu.cn
[3] Department of Mechanical Engineering, National University of Singapore, Singapore 117575, Singapore; seeram@nus.edu.sg
* Correspondence: y.dong@curtin.edu.au; Tel.: +61-8-9266-9055

Table S1. Physical and mechanical properties of TPU (MM-4520 grade) [27]

Physical properties		Mechanical properties		Thermal properties	
Melt viscosity (215°C; Pa·s)	3310	Young's modulus (MPa)	729	Glass transition temperature (˚C)	45
		Tensile strength at break (MPa)	41.4		
		Ultimate elongation (%)	600		
		Shore D hardness	72		

Table S2. Properties of HNTs [28]

Property	Value
Moisture content (%)	3.0
Specific gravity	2.55
pH (aqueous slurry at 20% solids)	3.5 – 4.5
Brunauer-Emmett-Teller (BET) surface area ($m^2 \cdot g^{-1}$)	25
Linear shrinkage (dried at 110°C) (%)	3.8
Modulus of rupture (dried at 110°C) (MPa)	2.9

Table S3. Data summary of filament extrusion parameters

Parameter	Setting
Front temperature (T_{front}) (°C)	165
Middle temperature (T_{mid}) (°C)	165
Back temperature (T_{back}) (°C)	165
Feed temperature (T_{feed}) (°C)	45
Extrusion speed (rpm)	50

Table S4. Data summary of abrasion resistance parameters

Parameter	Unit	Value
Sliding distance (S)	m	40
Rolling speed	rpm	40
Normal load	N	5
Abrasive sheet (corundum)	grid	60

Note: References [27] and [28] in Tables S1 and S2 are in accordance with the reference order in corresponding published research article.
